# A retrospective analysis of initial ventilator settings and lung mechanics in COVID-19 patients requiring mechanical ventilation

**DOI:** 10.62838/jccm-2026-0055

**Published:** 2026-10-30

**Authors:** Russell Vo, Uday Rallabhandi, Clark Walker, Ann Davis, Neil MacIntyre, John W. Hollingsworth

**Affiliations:** Texas Health Resources, Arlington, TX, USA; Texas Christian University Burnett School of Medicine, Fort Worth, TX, USA; Duke University School of Medicine, Durham, NC, USA

**Keywords:** Acute respiratory distress syndrome, ARDS, coronavirus disease 2019, COVID-19 pandemic, mechanical ventilation, and lung mechanics

## Abstract

**Background:**

During the COVID-19 pandemic, variability in lung mechanics challenged the conventional management of COVID-19-related hypoxemic respiratory failure, particularly on initial presentation. This study evaluated the association between patient characteristics, respiratory system mechanics, and ventilator settings in the first 24 hours of mechanical ventilation with mortality in a large cohort of patients with COVID-19-related hypoxemic respiratory failure.

**Methods:**

A multicenter, retrospective analysis of 1,563 mechanically ventilated patients with COVID-19 admitted to the ICU across sixteen hospitals in Texas between 2020 and 2023. Patient characteristics, respiratory system mechanics, and ventilator settings in the first 24 hours after intubation and mechanical ventilation were collected. All-cause in-hospital mortality was reported. Multivariable regression models were used to assess associations with mortality, with sequential addition of patient characteristics, severity of disease, and ventilator parameters.

**Results:**

Overall mortality in the study population was 54.4% with higher mortality was observed in patients with increasingly abnormal respiratory system mechanics and gas exchange. We also observed higher mortality associated with PP >30 cmH2O (64.5% vs 50.0%, p<0.001) and DP >14 cmH2O (56.8% vs 49.5%, p<0.007) and lower mortality associated with TV >8 cc/kg PBW compared to TV ≤8 cc/kg PBW (43.4% vs 59.4%, p<0.001) after multivariate regression analysis of covariates.

**Conclusion:**

Our retrospective observational, exploratory study illustrated associations between abnormal respiratory mechanics and gas exchange with mortality reported by many others in COVID-19 respiratory failure. The observation of lower mortality in patients receiving TVs >8 cc/kg PBW likely reflected severity of underlying disease and initial clinician judgment, thereby prioritizing minute ventilation needs in patients perceived to be at lower risk for ventilator-induced lung injury. Our study’s findings are limited to observations alone and should be cautiously interpreted, acknowledging the need for prospective, randomized control trials to further investigate whether or not there is an impact in mortality with TV >8 cc/kg PBW in otherwise safe mechanical ventilation.

## Introduction

The coronavirus disease 2019 (COVID-19) pandemic presented important challenges in the management of hypoxemic respiratory failure. Early reports described atypical physiologic patterns and variable lung compliance in patients with COVID-19, prompting debate about the applicability of conventional acute respiratory distress syndrome (ARDS) management principles [ 1, 2, 3, 4, 5]. Nevertheless, clinical practice largely adhered to the recommendations of ARDS Network (ARDSNet), emphasizing low tidal volume (TV <8ml/kg predicted body weight (PBW)) ventilation and plateau pressure (PP <30 cmH2O), and separately, driving pressure (DP <14 cmH2O) ( 6, 7, 8, 9, 10). These lung protective strategies aimed to minimize alveolar distension, barotrauma, and atelectrauma, thereby reducing the degree of ventilator-induced lung injury (VILI) [6, 7, 8, 11, 12].

Although prior studies have evaluated ventilator parameters measured at various time points in patients with COVID-19-related respiratory failure, large multicenter analyses evaluating the relationship between initial lung mechanics, ventilator settings, and mortality remain limited. Specifically, there is a lack of data on the relationship between the decision of clinicians regarding initial ventilator management and mortality outcomes in patients with COVID-19-related respiratory failure requiring admission to the intensive care unit (ICU). The aim of our research was focused on approaches to management on initial ICU presentation, which would offer unique insight into clinician thought processes at this critical time. Our specific objective examined the associations between patient characteristics, initial respiratory system data, and selection of initial ventilator settings in the first 24 hours of intubation, and in-hospital mortality.

## Methods

### Data Acquisition

A multicenter, retrospective observational study comprised of chart review across 16 Texas hospitals within the Texas Health Resources hospital system. Our total study cohort included 1,563 patients between 2020 to 2023. Inclusion criteria were individuals aged 18 to 98 years old, positive COVID-19 on reverse transcriptase polymerase chain reaction, and intensive care unit admission requiring invasive mechanical ventilation. We extracted data on patient mortality, demographics, comorbidities, date and time of intubation (from which year was derived), ventilator mode and settings, and partial pressure of oxygen to fraction of inspired oxygen (PaO2/FiO2) ratio. For the ventilator variables, only the first recorded value in the electronic medical record (EMR) within 24 hours of intubation and mechanical ventilation was collected into the data set and included in analysis; subsequent measurements were excluded. Data not documented within 24 hours of intubation and mechanical ventilation was excluded from the data set. Extracted ventilator settings were set TV in volume control modes, total respiratory rate (RR), FiO2, positive end expiratory pressure (PEEP), peak inspiratory pressure (PIP), and PP. Further ventilator parameters were calculated as follows: driving pressure (DP) as DP = PP-PEEP; static compliance (SC) as SC = TV/DP, and minute ventilation (MV) as MV = TV*RR. We calculated PBW (kg) = 50 + 2.3 × (height (inches) – 60) in males and PBW (kg) = 45.5 + 2.3 × (height (inches) – 60) in females (9). We did not collect data regarding sedation and are unable to factor this into the above data and calculations, specifically with regards to SC measurements. This study was approved by the Texas Christian University institutional review board #2022-207.

### Management Strategy

The participating hospitals had ventilator management protocols that include low TV and PP ventilation, and specific PEEP-FiO2 parameters consistent with lung protective strategies recommended by ARDSNet [9, 13, 14]. Importantly, clinician judgment was allowed for TV adjustments beyond the guideline recommended 4–8 cc/kg PBW to optimize patient comfort and MV while assuring DP and PP were <14 cmH2O and <30 cmH2O respectively.

### Statistical Analysis.

The primary outcome of the study was in-hospital mortality. Demographics, comorbidities, pandemic era, severity of disease (quantified by PaO2/FiO2 ratio and SC), and ventilator data, were analyzed with univariate analysis for the entire study population, dead, and alive cohorts (Table 1). Subgroup analysis of mortality was performed by stratifying the study population by widely accepted injury thresholds for PP >30 cmH2O, DP >14 cmH2O, and TV >8 cc/kg PBW (Figure 1) [ 15, 16, 17]. Out of our total study cohort of 1,563 patients, only 1,382 patients had data on both tidal volume and height. Therefore, 181 patients out of our total study cohort were excluded in our tidal volume subgroup analyses due to missing PBW data. Pandemic era was categorized as 2020, 2021, and 2022 and later based on epidemiologic phases of the COVID-19 pandemic. The previously presented baseline characteristics were re-analyzed with univariate analysis between cohorts of tidal volume and pandemic era, respectively (Supplemental Tables 1a and 1b). Incremental multivariable regression models were used to assess associations with mortality, with sequential addition of patient characteristics, severity of disease, and ventilator parameters (Figure 2; Supplemental Tables 2 and 3). Multicol-linearity was assessed using variance inflation factors (VIFs). A VIF >5 was considered indicative of significant multicollinearity. However, elevated VIFs (>5) for PP and DP were expected because these variables are physiologically and mathematically linked. Thus, the observed multicollinearity reflects the inherent relationship between PP and DP. TV was then analyzed as a binary variable (<8 cc/kg PBW vs >8 cc/kg PBW) in a multivariable regression model adjusting for patient characteristics and severity of disease (Table 2; Supplemental Table 4). White race and 2022+ were used as reference categories for race and pandemic era, respectively, in all regression models. Continuous variables were compared using Student’s t test for two-group comparisons and one-way ANOVA for three-group comparisons. Categorical variables were compared using Pearson’s chi-square tests. Statistical significance was determined by a cut off of p < 0.05. Confidence intervals (CI) were calculated using Wald approximation at a 95% confidence. Analyses were performed in Python using the pandas module for data manipulation, the statsmodels library for statistical analysis, and the matplotlib library for graphical display (18).

**Table 1.** Univariate analysis of patient characteristics, severity of disease, and initial ventilator data stratified by inhospital mortality.

	**Total n = 1563**	**Dead n = 850**	**Alive n = 713**	**P Value**
Demographics				
Age (years)	62.3	64.9	59.1	<0.001
BMI (kg/m^2^)	32.1	32.4	31.8	0.262
Sex (male)	60.5%	64.7%	55.4%	<0.001
Race				
Asian	3.1%	2.5%	3.9%	0.100
Black	13.9%	12.8%	15.1%	0.186
Hispanic	22.3%	22.6%	21.9%	0.737
White	52.6%	52.7%	52.5%	0.937
Other/Unknown	8.1%	9.4%	6.6%	0.042
Comorbidities				
Asthma	8.3%	7.0%	9.7%	0.054
CHF	21.4%	22.5%	20.2%	0.275
Cirrhosis	3.4%	3.2%	3.8%	0.503
COPD	10.3%	9.4%	11.3%	0.199
Diabetes	50.2%	52.6%	47.2%	0.034
Hypertension	52.5%	51.8%	53.3%	0.533
Renal Disease	7.9%	8.9%	6.8%	0.138
Smoking	41%	42.9%	39.2%	0.145
Severity of Disease				
PaO_2_/FiO_2_ Ratio	122.8	102.0	147.5	<0.001
Static Compliance (cc/cmH_2_O)	37.4	35.4	39.7	0.040
Ventilator Data				
FiO_2_	88.3%	92.2%	83.6%	<0.001
PEEP (cmH_2_O)	10.0	10.3	9.5	0.001
Plateau Pressure (cmH_2_O)	27.0	28.1	25.6	<0.001
Peak Inspiratory Pressure (cmH_2_O)	31.5	32.3	30.5	<0.001
Tidal Volume (mL/kg PBW)	7.8	7.5	8.1	<0.001
Minute Ventilation (L/min)	10.2	10.4	9.9	<0.001

**Figure 1.**
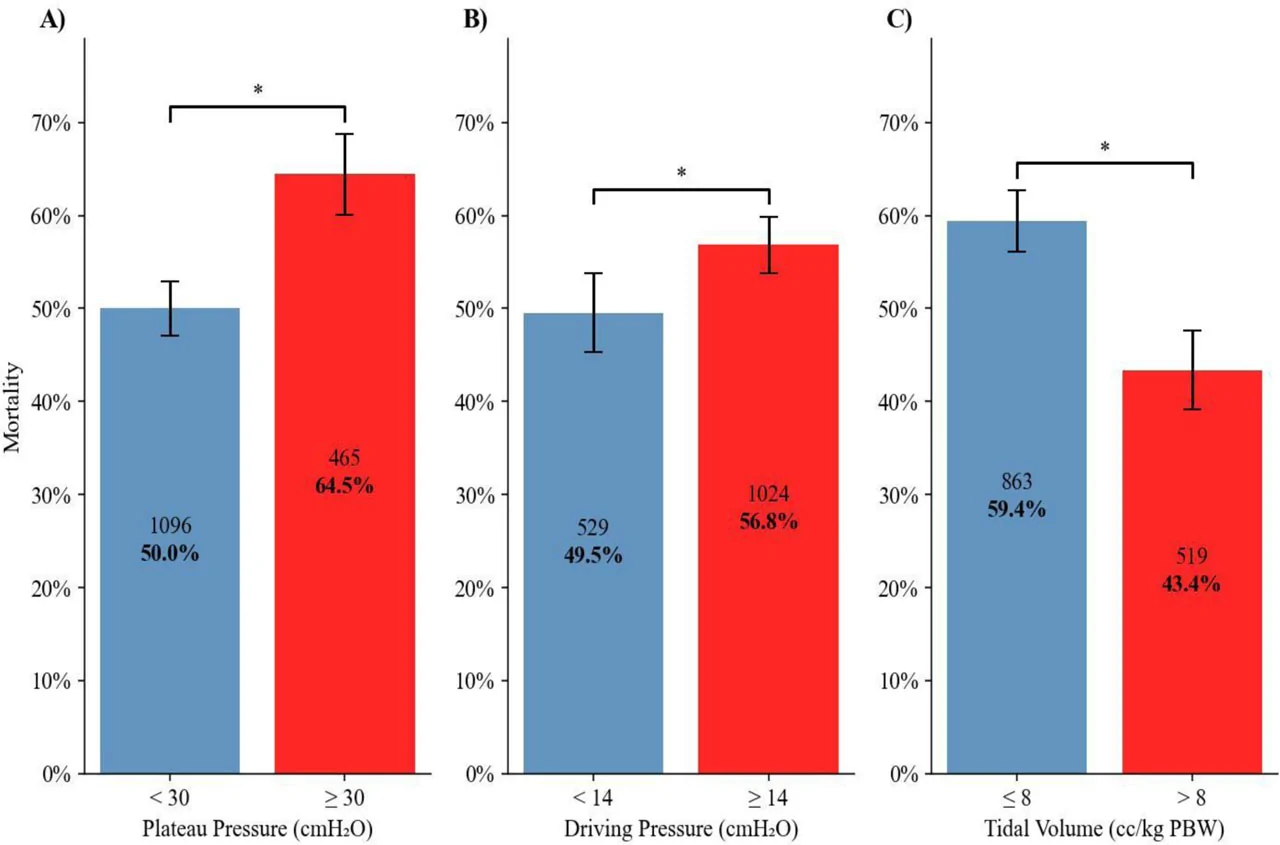
Unadjusted in-hospital mortality stratified by plateau pressure (A), driving pressure (B), and tidal volume (C). The error bars denote a 95% confidence interval and the asterisks indicate P value < 0.05.

**Figure 2.**
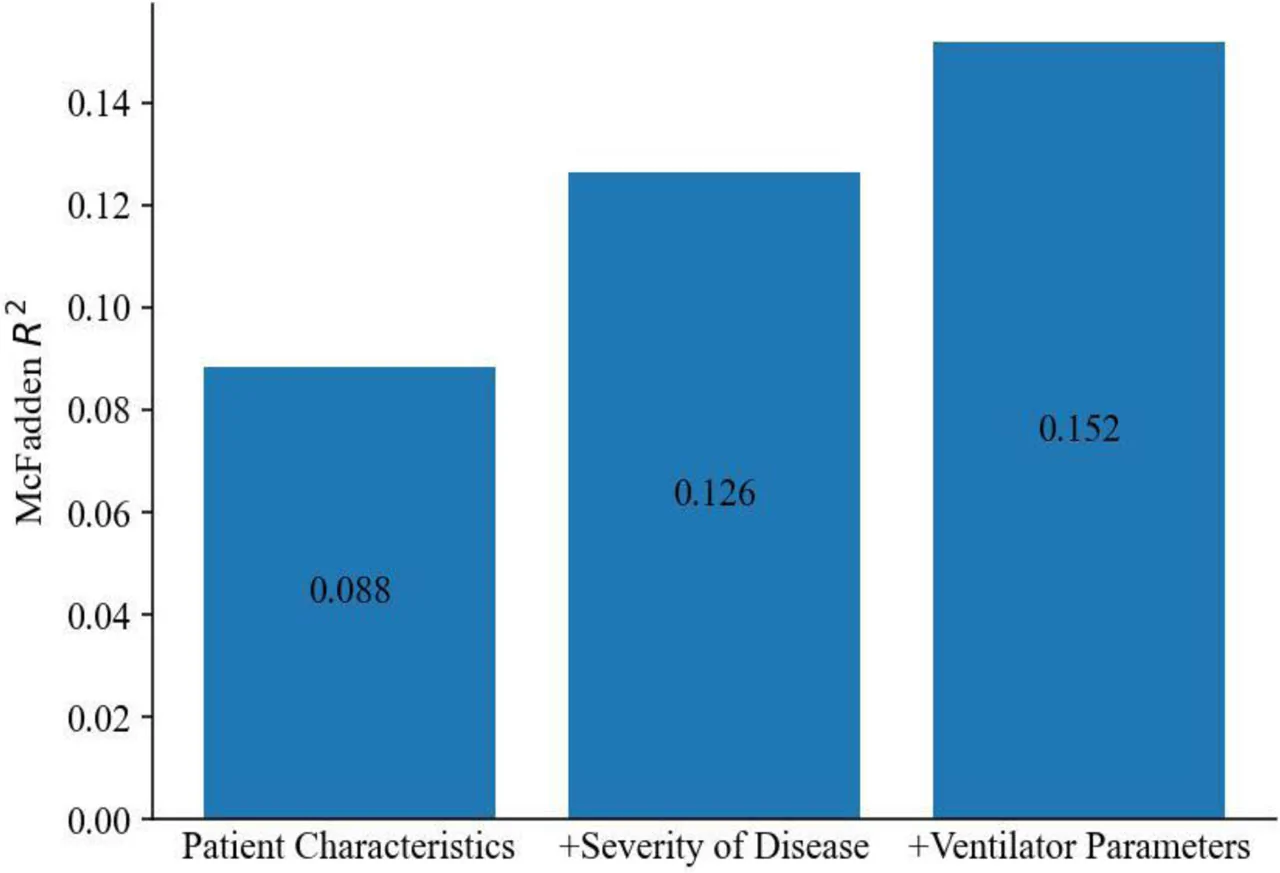
Incremental multivariable regression models with sequential addition of patient characteristics, severity of disease, and ventilator parameters to determine goodness-of-fit.

## Results

### Demographics.

The total study population included 1,563 patients, which comprised of 945 (60.5%) males and 618 (39.5%) females with a mean age of 62.3 years and mean body mass index of 32.1 kg/m^2^ (Table 1). The study population was comprised of majority White, non-Hispanic (52.6%) followed by Hispanic (22.3%), Black (13.9%), Asian (3.1%), and other/unknown (8.1%). The most prevalent comorbidities included hypertension (52.5%), diabetes mellitus (50.2%), history of smoking (41.0%), congestive heart failure (CHF) (21.4%), and chronic obstructive pulmonary disease (COPD) (10.3%).

### Mortality Predictors.

The observed in-hospital mortality in the study population was 850 patients (54.4%). Univariate analysis of demographics, comorbidities, severity of disease, and ventilator parameters are presented in Table 1. The most common ventilator modes were volume assist control (74.1%), pressure-regulated volume control (15.7%), pressure assist control (6.6%), and other (3.6%). Consistent with established injury thresholds, higher PP (>30 cmH2O) and DP (>14 cmH2O) within 24 hours of intubation were associated with higher mortality (Figure 1A and 1B). In contrast, higher TV (>8 cc/kg PBW) was associated with lower mortality (p < 0.001) (Figure 1C).

Three incremental multivariable regression models were constructed to determine the contribution of subsequent variable blocks to mortality (Figure 2; Supplemental Table 2). The base model of patient characteristics including demographics, comorbidities, and pandemic era demonstrated a McFadden R^2^ of 0.088. The addition of severity of disease and select ventilator parameters (PP, DP, TV) further improved model fit (Δ McFadden R^2^ = 0.038 and 0.026, respectively), with statistical significance determined by likelihood ratio testing (p < 0.001 for both comparisons). The final model is presented in Supplemental Table 3.

Given the unexpected observed association between higher TV (>8 cc/kg PBW) and lower mortality, an additional multivariable regression model was constructed with TV as a binary variable (≤8 vs >8 cc/kg PBW) (Table 2; Supplemental Table 4). Higher TV was associated with lower odds of death (OR 0.57, 95% CI 0.43–0.77) after adjustment for patient characteristics, severity of disease, and pandemic era.

**Table 2:** Multivariate logistic regression model of in-hospital mortality with tidal volume as a binary variable, adjusting for patient characteristics and severity of disease. Adjusted odds ratios (OR) and their 95% confidence interval are reported.

**Variable**	**Adjusted OR (95% CI)**
Tidal Volume (>8 cc/kg PBW)	0.57 (0.43–0.77)
Age (per 10 years)	1.54 (1.39–1.72)
BMI (per 5 kg/m^2^)	1.15 (1.07–1.25)
Mid Pandemic (2021)	1.90 (1.31–2.78)
COPD	0.55 (0.35–0.86)
Renal Disease	1.80 (1.04–3.10)
PaO_2_/FiO_2_ ratio (per 50 mmHg)	0.83 (0.78–0.88)

## Discussion

Patient characteristics and severity of lung disease contributed most to model fit, underscoring the complex interplay between patient physiology, severity of underlying lung disease, and outcomes in COVID-19-associated respiratory failure. However, incremental modeling demonstrated ventilator parameters also contributed significantly to model fit, reaffirming the importance of lung protective strategies, presumably to minimize VILI from alveolar overdistension and barotrauma and in the setting of disease severity (9, 11, 12). Consistent with prior literature, elevated PP (>30 cmH2O) and DP (>14 cmH2O) were associated with increased mortality [9, 14]. Unexpectedly, our analysis observed an association between higher TV (>8 cc/kg PBW) within 24 hours of intubation and lower mortality. While it is possible that large TV selection was a random clinical decision that revealed a benefit in COVID-19 patients, we believe instead that it more likely reflects clinician judgment prioritizing patient comfort and minute ventilation over rigid TV limits in patients with otherwise safe ventilator settings. Supporting this interpretation is the observation that high TVs appeared to be applied preferentially to patients with better gas exchange (P/F ratio) and “safe” (i.e. below injury thresholds) values for PP and DP (Supplemental Table 2). In other words, we believe clinicians in the first 24 hours of mechanical ventilation for patients with severe respiratory failure were faced with achieving a balance between adequate ventilatory support and optimizing lung protection. Prioritization of ventilator settings to support gas exchange and patient comfort might then drive a decision for a higher TV if severity of lung disease allows and other markers of lung injury risk (i.e. PP and DP) are acceptable. Our study is limited by its retrospective observational design and cannot establish causality; although TV >8 ml/kg PBW were observed to be independently associated with lower mortality, this is strictly an observation alone.

The strength of the study lies in its large sample size, focus on initial measurements in the first 24 hours of intubation, and inclusion of patients from a broad network of community hospitals, which enhances its external validity. However, there are several major limitations that warrant mention. The retrospective, observational study design precludes any causal inference between ventilator parameters and mortality. The use of EMR data also carries inherent variability and incomplete documentation, despite manual verification of outlier values (TV <4 cc/kg PBW and>15 cc/kg PBW). Several variables had missing data including demographics (<0.3%), medical comorbidities (<3.3%), ventilator data (11.6%) and severity of disease (<8%) and thus were excluded, thereby precluding further objective analysis of severity of lung disease and may be potential confounders. Because the data collected and analyzed was from a single time point (first 24 hours of mechanical ventilation), changes occurring during the course of illness could not be studied. Clinician decision-making regarding ventilator management could not be standardized or inferred from our retrospective review of the EMR. We did not collect data regarding sedation or use of paralytics, which may impact the interpretation of our initial data collected. Additionally, we excluded data regarding laboratory values and treatments received from our data analysis due to high missingness rate of up to 92.3%. Data was obtained from multiple hospitals in this multicenter study design, which opens the study to intercenter variability. Lastly, there is residual confounding, particularly by disease severity and clinician decision-making, as well as confounding by indication, as ventilator settings likely reflect clinician assessment of patient’s severity of underlying disease.

## Conclusions

Retrospective, observational, exploratory study of a large multicenter database of severe COVID-19 respiratory failure patients observed anticipated associations regarding adverse effects of abnormal lung function on outcome. The analysis also revealed that clinician management across a large network of hospitals generally appeared to follow lung protective guidelines. Interestingly, higher initial TVs were strictly observed to be associated with lower mortality in our study after adjusting for confounders including patient characteristics, pandemic era, severity of disease, and ventilator data. Given the nature of the study design, this association should be cautiously interpreted and does not imply causality. We suspect this reflects clinician behavior of balancing minute ventilation needs versus lung protection and severity of underlying disease, which may explain our study’s findings. Further studies including prospective, randomized control trials are needed to determine whether the observed associations reflect differences in patient severity of underlying disease, clinician ventilator selection, or other unmeasured confounders.

## Supplementary Material

Supplementary Material Details
