# Supplementary material for: A retrospective analysis of initial ventilator settings and lung mechanics in COVID-19 patients requiring mechanical ventilation
Source: J Crit Care Med (Targu Mures). 2026 Oct 30;12(4):636–42. doi: 10.62838/jccm-2026-0055 (PMC13752166; doi:10.62838/jccm-2026-0055)
Supplement: Supplementary file 1 [file jccm-2026-0055-supplementary.pdf]

Supplemental Table 1a. Univariate analysis of patient characteristics, severity of disease, and initial ventilator data stratified by tidal volume ventilation: ≤8 cc/kg and >8 cc/kg of predicted body weight (PBW).

	≤8 cc/kg PBW	>8 cc/kg PBW	P Value
	n = 863	n = 519	
Mortality	59.4%	43.4%	<0.001
Demographics			
Age (years)	62.5	62.3	0.827
BMI (kg/m^2)	31.5	32.2	0.149
Sex (male)	73.1%	38.3%	<0.001
Race			
Asian	2.4%	4.2%	0.065
Black	14.3%	14.6%	0.853
Hispanic	19.2%	27.4%	0.002
White	55.6%	45.5%	<0.001
Other/Unknown	8.5%	8.3%	0.914
Comorbidities			
Asthma	8.6%	7.8%	0.587
CHF	21.9%	22.9%	0.662
Cirrhosis	3.1%	4.5%	0.175
COPD	9.0%	13.5%	0.012
Diabetes	49.1%	51.4%	0.568
Hypertension	50.2%	53.7%	0.395
Renal Disease	7.3%	9.6%	0.152
Smoking	44.1%	38.4%	0.114
Severity of Disease			
PaO_2/FiO_2 Ratio	133.6	179.2	<0.001
Static Compliance (cc/cmH_2O)	37.7	37.4	0.901
Ventilator Data			
FiO_2	89.1%	85.9%	0.006
PEEP (cmH_2O)	10.0	9.4	0.006
Plateau Pressure (cmH_2O)	26.2	26.8	0.124
Peak Inspiratory Pressure (cmH_2O)	30.9	32.4	0.001
Tidal Volume (mL/kg PBW)	6.7	9.6	<0.001
Minute Ventilation (L/min)	10.2	10.2	0.885

Supplemental Table 1b. Univariate analysis of in-hospital mortality, patient characteristics, severity of disease, and initial ventilator data stratified by pandemic era: 2020-2021, 2021-2022, and 2022 and beyond.

	2020 n = 492	2021 n = 772	2022+ n = 299	P Value
Mortality	50.8%	62.0%	40.5%	<0.001
Demographics				
Age (years)	62.8	60.8	65.4	<0.001
BMI (kg/m^2)	31.8	33.2	29.8	<0.001
Sex (male)	66.1%	59.8%	52.8%	0.001
Race				
Asian	3.3%	2.3%	5.0%	0.076
Black	15.2%	13.2%	13.4%	0.572
Hispanic	31.1%	21.0%	11.0%	<0.001
White	39.0%	55.4%	67.6%	<0.001
Other/Unknown	11.4%	8.0%	3.0%	<0.001
Comorbidities				
Asthma	7.6%	8.8%	8.0%	0.725
CHF	21.2%	17.9%	30.4%	<0.001
Cirrhosis	2.7%	2.9%	6.0%	0.024
COPD	8.8%	8.4%	17.1%	<0.001

	2020 n = 492	2021 n = 772	2022+ n = 299	P Value
Diabetes	55.7%	49.6%	42.8%	0.002
Hypertension	56.3%	51.4%	49.2%	0.107
Renal Disease	6.7%	8.0%	9.7%	0.327
Smoking	41.8%	39.5%	44.5%	0.326
Severity of Disease				
PaO_2/FiO_2 Ratio	141.9	127.8	212.3	<0.001
Static Compliance (cc/cmH_2O)	39.4	36.6	36.9	0.504
Ventilator Data				
FiO_2	87.8%	91.6%	80.5%	<0.001
PEEP (cmH_2O)	9.9	10.6	8.5	<0.001
Plateau Pressure (cmH_2O)	27.1	27.8	24.8	<0.001
Peak Inspiratory Pressure (cmH_2O)	32.1	31.9	29.7	0.002
Tidal Volume (mL/kg PBW)	8.0	7.6	7.9	<0.001
Minute Ventilation (L/min)	10.0	10.5	9.6	<0.001

Supplemental Table 2. Performance measures of the incremental multivariable regression models.

Model	LL	AIC	McFadden R2	McFadden ΔR2	AUC	LR P vs Previous
Patient Characteristics	-700.2	1436.4	0.088	-	0.700	-
+Severity of Disease	-671.0	1382.1	0.126	0.038	0.775	<0.001
+Ventilator Parameters	-651.7	1349.4	0.152	0.026	0.752	<0.001

Supplemental Table 3. Final incremental multivariate logistic regression model of in-hospital mortality with individualized patient characteristics, severity of disease, and ventilator parameters (covariates) and in-hospital mortality. Adjusted odds ratios (OR) and their 95% confidence intervals are reported. Variance inflation factors (VIF) analysis performed to quantify the degree of regression coefficient inflation (multicollinearity).

Variables	Adjusted OR (95% CI)	P Value	VIF
Tidal Volume (cc/kg PBW)	0.83 (0.77–0.91)	<0.001	1.295
PaO_2/FiO_2 Ratio (per 50 mmHg)	0.86 (0.81–0.92)	<0.001	1.285
Static Compliance (per 10 cc/cmH_2O)	1.02 (0.98–1.06)	0.349	1.439
Age (per 10 years)	1.59 (1.43–1.78)	<0.001	1.272
BMI (per 5 kg/m^2)	1.14 (1.05–1.23)	0.002	1.266
Sex (male)	1.27 (0.93–1.73)	0.127	1.340
Asian	1.23 (0.55–2.75)	0.622	1.071
Black	0.92 (0.61–1.38)	0.691	1.198
Hispanic	1.22 (0.84–1.76)	0.293	1.368
Other/Unknown Race	1.64 (0.97–2.78)	0.064	1.151
Early Pandemic (2020)	1.26 (0.84–1.89)	0.260	2.071
Mid Pandemic (2021)	1.81 (1.23–2.64)	0.002	2.062
Asthma	0.68 (0.42–1.10)	0.112	1.075
CHF	0.97 (0.69–1.37)	0.880	1.171
Cirrhosis	1.19 (0.59–2.44)	0.625	1.022
COPD	0.56 (0.36–0.88)	0.012	1.154
Diabetes	1.03 (0.78–1.35)	0.848	1.109
Hypertension	0.77 (0.58–1.01)	0.062	1.145
Renal Disease	1.78 (1.03–3.09)	0.040	1.207
Smoking	1.04 (0.78–1.37)	0.791	1.110
Driving Pressure (cmH_2O)	1.01 (0.96–1.06)	0.708	5.455
Plateau Pressure (cmH_2O)	1.05 (1.01–1.10)	0.028	5.545

Supplemental Table 4. Multivariate logistic regression model of in-hospital mortality with tidal volume as a binary variable adjusting for individualized patient characteristics and severity of disease (covariates). Adjusted odds ratios (OR) and their 95% confidence interval are reported. Variance inflation factors (VIF) analysis performed to quantify the degree of regression coefficient inflation (multicollinearity).

Variables	Adjusted OR (95% CI)	P Value	VIF
Tidal Volume (>8 cc/kg PBW)	0.57 (0.43–0.77)	<0.001	1.233
PaO_2/FiO_2 Ratio (per 50 mmHg)	0.83 (0.78–0.88)	<0.001	1.171
Static Compliance (per 10 cc/cmH_2O)	0.97 (0.94–1.01)	0.118	1.059
Age (per 10 years)	1.54 (1.39–1.72)	<0.001	1.268
BMI (per 5 kg/m^2)	1.15 (1.07–1.25)	<0.001	1.195
Sex (male)	1.26 (0.94–1.71)	0.125	1.298
Asian	1.15 (0.51–2.56)	0.738	1.068
Black	0.90 (0.60–1.34)	0.593	1.192
Hispanic	1.17 (0.82–1.67)	0.380	1.339
Other/Unknown Race	1.61 (0.96–2.71)	0.071	1.146
Early Pandemic (2020)	1.36 (0.92–2.03)	0.125	2.043
Mid Pandemic (2021)	1.90 (1.31–2.76)	<0.001	1.993
Asthma	0.68 (0.42–1.10)	0.113	1.075
CHF	0.98 (0.69–1.37)	0.890	1.168
Cirrhosis	1.18 (0.57–2.43)	0.656	1.022
COPD	0.55 (0.35–0.86)	0.009	1.151
Diabetes	1.03 (0.79–1.36)	0.808	1.107
Hypertension	0.78 (0.59–1.03)	0.077	1.140
Renal Disease	1.80 (1.04–3.10)	0.036	1.203
Smoking	1.03 (0.78–1.35)	0.857	1.110
